# Characterization of the clinical course of early‐onset severe hemolytic disease of the fetus and newborn

**DOI:** 10.1002/pmf2.70352

**Published:** 2026-07-12

**Authors:** Dick Oepkes, Kenneth J. Moise, Enrico Lopriore, Eleonor Tiblad, E. J. T. (Joanne) Verweij, Rory Windrim, Pranav Pandya, Robert M. Silver, Stephen P. Emery, John Smoleniec, Olga Ocón‐Hernández, Rachel K. Morris, Russell S. Miller, James B. Streisand, Diane D. Harrison, Valerie Smith, Lisa B. Schwartz, May Lee Tjoa, Leona E. Ling, Shumyla Saeed‐Khawaja, Yosuke Komatsu

**Affiliations:** ^1^ Department of Obstetrics Leiden University Medical Center Leiden The Netherlands; ^2^ Dell Medical School The University of Texas at Austin Austin Texas USA; ^3^ Comprehensive Fetal Care Center at Dell Children's Medical Center Austin Texas USA; ^4^ Department of Pediatrics Leiden University Medical Center Leiden The Netherlands; ^5^ Clinical Epidemiology Division Department of Medicine, Solna Karolinska Institutet Stockholm Sweden; ^6^ Ontario Fetal Centre Mount Sinai Hospital University of Toronto Toronto Ontario Canada; ^7^ Fetal Medicine Unit University College London Hospitals NHS Foundation Trust London UK; ^8^ Department of Obstetrics and Gynecology University of Utah School of Medicine Salt Lake City Utah USA; ^9^ Department of Obstetrics Gynecology, and Reproductive Sciences UPMC Magee‐Womens Hospital Pittsburgh Pennsylvania USA; ^10^ Feto‐Maternal Unit Liverpool Hospital Liverpool New South Wales Australia; ^11^ Fetal Medicine Unit San Cecilio University Hospital and ibs.GRANADA Granada Spain; ^12^ Fetal Medicine Centre Birmingham Women's and Children's NHS Foundation Trust Birmingham UK; ^13^ Department of Applied Health Sciences, School of Health Sciences, College of Medicine and Health University of Birmingham Birmingham UK; ^14^ Division of Maternal Fetal Medicine Department of Obstetrics and Gynecology Columbia University Irving Medical Center New York New York USA; ^15^ Streisand Biomedical Consulting Wayland Massachusetts USA; ^16^ Johnson & Johnson Spring House Pennsylvania USA; ^17^ Johnson & Johnson Raritan New Jersey USA; ^18^ Johnson & Johnson Cambridge Massachusetts USA

**Keywords:** fetal/neonatal anemia, HDFN, hemolytic disease of the fetus and newborn, intrauterine blood transfusion, intravenous immunoglobulins, neonatal/postnatal outcome, plasmapheresis

## Abstract

**Introduction:**

Severe fetal anemia developing at ≤24 weeks gestational age (GA) in pregnancies complicated by early‐onset severe hemolytic disease of the fetus and newborn (EOS‐HDFN) is managed by intrauterine erythrocyte transfusions (IUTs). Evidence of off‐label intravenous immunoglobulin (IVIG) for preventing/reducing fetal hydrops, fetal anemia, and IUT(s) remains limited to retrospective studies and case series. The prospective, global, multicenter, observational CLARITY study characterized standard‐of‐care management and outcomes for high‐risk pregnancies complicated by EOS‐HDFN and their offspring at expert referral centers.

**Methods:**

CLARITY enrolled individuals with singleton pregnancies at ≥8 weeks GA and high risk for EOS‐HDFN, defined by prior severe fetal anemia, fetal hydrops, or stillbirth at ≤24 weeks GA with indicative fetal/placental HDFN pathology, and current titers ≥32 anti‐Rhesus D (RhD) or ≥4 anti‐Kell with a confirmed antigen‐positive fetus.

**Results:**

Of 15 pregnant participants, two (13%) achieved the primary endpoint of a live birth at ≥32 weeks GA without IUT, indicating the absence of clinically relevant fetal anemia and low disease burden. Fourteen (93%) pregnancies resulted in a live birth (median GA, 36^2/7^ weeks; range, 31^4/7^–38^0/7^), including seven (50%) preterm births and 12 (86%) cesarean deliveries. Thirteen (87%) participants required IUTs (median IUTs/pregnancy, 4; range, 1–11), with median GA at first IUT of 24^0/7^ weeks (range, 13^0/7^–28^2/7^). One (7%) fetal death occurred at 16^5/7^ weeks, following early IUT (13^0/7^ weeks) and serial IVIG treatment (13^1/7^–14^6/7^ weeks). IUT complications occurred in four (31%) pregnant participants who received IUTs. In an exploratory, post hoc analysis, eight (53%) pregnant participants received IVIG starting between 10^6⁄7^ and 19^3⁄7^ weeks (median administrations/pregnancy, 12.5; range, 1–20); IVIG‐related complications were reported in three (38%), and two (25%) met the primary endpoint. Of 14 live‐born neonates followed through 12 weeks of life, three (21%) received exchange transfusion(s), nine (64%) received simple transfusion(s) (one required both types of transfusions), while three (21%) required no transfusions (one required no IUTs during the pregnancy).

**Conclusion:**

Outcomes of this prospective study of high‐risk EOS‐HDFN pregnant participants and their offspring are consistent with previous retrospective studies, indicating a high risk of early IUT, neonatal transfusions, and fetal/neonatal morbidity and mortality. The results underscore the unmet medical need for effective treatments for EOS‐HDFN.

**Trial registration:**

ClinicalTrials.gov Identifier: NCT03755128.

## 1 INTRODUCTION

Severe hemolytic disease of the fetus and newborn (HDFN) is a life‐threatening condition occurring when mismatched maternal immunoglobulin G (IgG) alloantibodies cross the placenta during pregnancy and bind to antigens on fetal red blood cells (RBCs), thereby causing RBC destruction and fetal anemia [1]. While Rhesus D (RhD) prophylaxis has significantly reduced the incidence of severe HDFN, prophylaxis programs do not adequately prevent all cases of RhD sensitization [1, 2, 3, 4, 5, 6, 7]. The condition also persists as a rare disease due to non‐RhD alloimmunization [1, 2, 3, 4, 5, 6, 7].

Early‐onset severe HDFN (EOS‐HDFN) is the most severe and rare form of this condition, where fetal anemia begins at ≤24 weeks gestational age (GA). In pregnancies complicated by EOS‐HDFN, rescue from fetal anemia typically involves intrauterine transfusion (IUT) of RBCs, performed intravascularly (IVT) at GA ≥20 weeks, or via intraperitoneal access (IPT) at <20 weeks. Due to disease sequelae and procedural risks associated with IUT, EOS‐HDFN can carry an increased risk of complications (e.g., fetal bradycardia) and fetal demise (17%–24%) [8, 9, 10, 11, 12, 13, 14, 15]. Following antenatal fetal anemia, neonatal hyperbilirubinemia requires standard or intensive phototherapy, with 24% of neonates requiring transfusions; neonatal anemia results in 63%–88% of neonates receiving simple transfusions (also known as top‐up transfusions) [12, 16, 17]. Finally, the risk of EOS‐HDFN or fetal anemia recurrence in a subsequent fetal antigen‐positive pregnancy is estimated to be 80%–100% [9, 10, 18, 19, 20, 21]. Thus, subspecialized medical management at HDFN referral centers is required to minimize the risk of fetal and neonatal morbidity and mortality.

Management of EOS‐HDFN generally follows guidelines for frequent fetal monitoring for anemia by middle cerebral artery peak systolic velocity (MCA‐PSV) by Doppler ultrasound and for signs of anemia‐induced cardiomegaly, hepatosplenomegaly, and fetal hydrops [22, 23]. When the MCA‐PSV is >1.5 multiples of the median (MoM), cordocentesis is indicated to confirm moderate to severe fetal anemia that would require an IUT [22]. The timing of subsequent IUTs is determined by estimated decrease in hematocrit or by MCA‐PSV surveillance. Repeat IUTs are typically required to achieve late preterm or term birth [18, 24]. In EOS‐HDFN cases occurring at <20 weeks GA, IPTs are performed until cordocentesis is possible.

Due to its rarity, EOS‐HDFN management and outcomes have been only described in retrospective studies and case series [9, 10, 21, 25]. Given the risk of poor antenatal/neonatal outcomes and high unmet medical need, these reports often highlight outcomes of unapproved treatments (e.g., intravenous immunoglobulin [IVIG] or plasmapheresis) used by some HDFN referral centers to decrease the likelihood of EOS‐HDFN manifestations [9, 10, 21, 25]. Recent large retrospective studies suggest that IVIG may decrease the risk of fetal hydrops, delay the onset of fetal anemia, and, in a minority of cases, prevent the need for IUTs in EOS‐HDFN [9, 10, 21]. A meta‐analysis of retrospective studies showed similar results [26]. The outcomes are reported here of the CLARITY study, which is a prospective, global, multicenter, observational study that provides insight into the current management and outcomes of pregnancies at high risk of fetal anemia and EOS‐HDFN receiving care at global HDFN referral centers. Furthermore, due to its similar study design, CLARITY provides context for the previously published clinical outcomes of the open‐label, single‐arm, Phase 2 UNITY study of nipocalimab, a novel antibody therapeutic targeting the neonatal Fc receptor (FcRn), in pregnant individuals at high risk for EOS‐HDFN [20].

## 2 MATERIALS AND METHODS

### 2.1 Ethics

This study was conducted in compliance with Good Clinical Practice and the Declaration of Helsinki, as well as applicable local regulations. All pregnant participants and legal representatives for offspring provided written informed consent before enrollment. The study protocol was approved by the appropriate institutional review board/independent ethics review committees at each site (details are provided in Table S1). The study was a noninterventional trial designed in alignment with the UNITY trial of nipocalimab (e.g., study eligibility criteria, study duration, and assessments) [20] to help contextualize those results. Study sites received financial support from the sponsor to conduct the study. Of the 18 centers that participated in CLARITY, 14 also participated in UNITY. However, the two studies were independent, and each followed standard procedures with rigorous oversight to minimize bias.

### 2.2 Participants

Eligible participants were aged ≥18 years, pregnant with a single fetus with an estimated GA ≥8 weeks based on ultrasound or last menstrual period (no upper limit on GA at enrollment), and were at high risk of EOS‐HDFN, as defined by (1) having a previous qualifying pregnancy with ≥1 of the following at ≤24 weeks GA: severe fetal anemia (hemoglobin <0.55 MoM for GA at cordocentesis) [27], fetal hydrops with MCA‐PSV MoM ≥1.5, or previous stillbirth with fetal/placental pathology indicative of HDFN; and (2) having titers for maternal anti‐D ≥32 or anti‐K alloantibodies ≥4 and with confirmation of an antigen‐positive fetus (i.e., paternal homozygous D or K antigen, cell‐free fetal DNA or fetal antigen status as documented by maternal amniocentesis consistent with a D‐positive or K‐positive fetus) in the current pregnancy. Participants with preeclampsia in the current or previous pregnancy or who were participating in a clinical trial for an investigational therapy for HDFN were excluded to align with UNITY exclusion criteria [20]. Participants were enrolled from the same expert HDFN referral centers as the UNITY trial [20]; one participant who had enrolled in the CLARITY study was later enrolled in the UNITY study [20] in a subsequent pregnancy.

### 2.3 Study design

Participants underwent clinical assessments and received standard of care for EOS‐HDFN (i.e., IUTs) per local practice, as determined by their physician. The study included a screening period, standard‐of‐care treatment period, 6‐month postnatal follow‐up period for pregnant participants, and 1‐year follow‐up period for all neonates/infants (Figure S1). Neonates/infants received standard of care for EOS‐HDFN up to 3 months of age.

### 2.4 Outcomes

The primary endpoint was the proportion of pregnant participants with a live birth at ≥32 weeks GA without an IUT. This endpoint was selected as a marker of a lack of clinically relevant fetal anemia and lower disease burden, by capturing both (1) avoidance of invasive fetal rescue therapy (IUT), which indicates clinically significant fetal anemia and carries procedural risks, and (2) achievement of GA milestone (≥32 weeks) associated with substantially lower prematurity‐related neonatal morbidity. Antenatal/perinatal endpoints included the percentage of live births, GA at first IUT, MCA‐PSV from baseline to first IUT, hemoglobin and hematocrit values prior to first IUT, number of IUTs required, percentage of participants without an IUT before 24 weeks GA, percentage of participants with fetal hydrops (severe edema in the skin and serous cavities diagnosed via ultrasound), and GA at delivery. The percentage of pregnant participants receiving plasmapheresis and/or IVIG was collected as a post hoc analysis. Complications associated with IUT (e.g., fetal bradycardia, cord bleeding, infection, preterm premature rupture of membranes, preterm delivery, emergency cesarean delivery, fetal demise, other) and with IVIG were also reported prospectively. However, consistent with the observational, natural history design of the study, complications of IUT and IVIG treatment were not graded for severity or calculated per intervention.

Postnatal endpoints included the percentage of neonates requiring phototherapy, number of hours of phototherapy, percentage of neonates requiring exchange transfusions, number of exchange transfusions required by neonates, percentage of neonates/infants requiring simple transfusions in the first 12 weeks of life, and number of simple transfusions required by neonates/infants in the first 12 weeks of life. Exploratory outcomes included duration of hospitalization and neonatal intensive care unit (NICU) admission.

### 2.5 Statistical analyses

The sample size of 15 evaluable pregnant participants was based on medical considerations and the rarity of the EOS‐HDFN study population. This study was designed to be descriptive and hypothesis‐generating; therefore, no formal statistical power calculations or formal hypothesis testing were performed for any outcome. Summary statistics are presented, with 95% Clopper–Pearson confidence intervals (CIs) reported for all at‐risk pregnancies for each endpoint as appropriate.

## 3 RESULTS

### 3.1 Participants

From August 2019 to September 2023, 19 pregnant individuals were screened, and 15 were enrolled at nine national and regional HDFN referral centers in seven countries (Figure 1). All 15 pregnant participants were included in the maternal analyses; 12 completed the study, and three discontinued early (one fetal loss and two lost to follow‐up after delivery). Fourteen live births and one fetal loss occurred. Of the 14 neonates/infants included in the analyses, 11 completed the study; the three who discontinued were lost to follow‐up.

**FIGURE 1:**
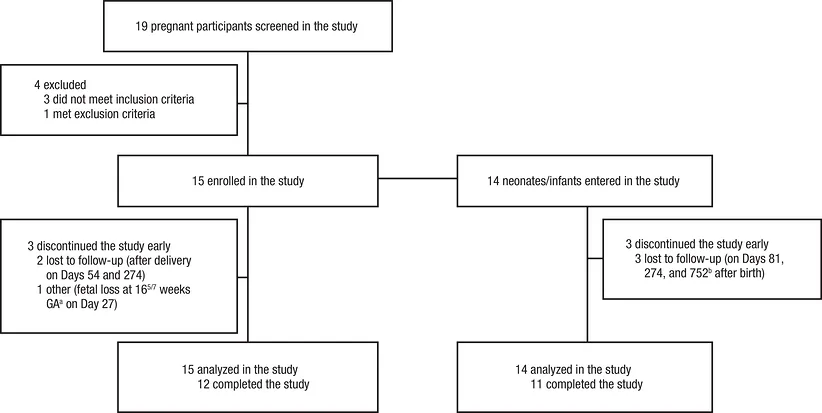
Study disposition of pregnant participants and neonates/infants. Participants with EOS‐HDFN were screened for inclusion in the study, and reasons for exclusion or early discontinuation for pregnant participants and their neonates/infants are shown. EOS‐HDFN, early‐onset severe hemolytic disease of the fetus and newborn; GA, gestational age. ^a^Based on GA at informed consent. ^b^The loss to follow‐up at Day 752 is most likely a record/transcription error (an administrative/last‐contact date captured outside the protocol‐defined window).

Characteristics of pregnant participants and their most recent qualifying pregnancies are described in Table 1 and Table S2. Eleven (73%) were White and 14 (93%) had anti‐D alloantibodies. Six (40%) pregnant participants with anti‐D alloantibodies also presented with ≥1 other alloantibody type, including anti‐C alone (*n* = 3), anti‐C and ‐E (*n* = 2), and anti‐E alone (*n* = 1). One (7%) had anti‐Kell alloantibodies. The median age was 36.0 years (range, 29–42) and the median GA was 22^3/7^ weeks (range, 13^0/7^–35^5/7^) at the time of informed consent.

**TABLE 1:** Demographic and baseline characteristics of pregnant participants and their qualifying pregnancies.

	Participant ID															
**Characteristic**	1	2	3	4	5	6	7	8	9	10	11	12	13	14	15	Total (*N *= 15)
Characteristics at enrollment																
Age, median (range), years	31	42	40	42	33	38	36	40	36	37	36	30	36	31	29	36.0 (29–42)
White race, *n* (%)	No	Yes	Yes	Yes	Unknown	Yes	Yes	Yes	Not reported	Yes	No	Yes	Yes	Yes	Yes	11 (73)
RhD alloantibody type,^a^ *n* (%)	Yes	Yes^a^	No	Yes	Yes^a^	Yes^a^	Yes	Yes	Yes	Yes	Yes	Yes^a^	Yes	Yes^a^	Yes^a^	14 (93)
GA at informed consent, median (range)	28^5/7^	13^0/7^	24^2/7^	18^1/7^	18^6/7^	18^2/7^	35^5/7^	16^3/7^	22^3/7^	24^6/7^	24^3/7^	15^0/7^	14^4/7^	27^0/7^	22^5/7^	22^3/7^ (13^0/7^–35^5/7^)
Time between study pregnancy and last qualifying pregnancy,^b^ median (range), years	3.0	14.6	6.8	12.6	8.4	1.1	1.0	4.6	NA	1.9	0.3	3.1	7.1	0.3	2.0	3.0 (0.3–14.6)
Time between study pregnancy and last HDFN‐affected pregnancy,^c^ median (range), years	3.0	14.6	2.2	12.6	8.4	1.1	1.0	4.6	NA	1.9	0.3	3.1	7.1	0.3	0.5	2.6 (0.3–14.6)
Most recent qualifying pregnancy																
Live birth, *n* (%)	Yes	No	No	No	Yes	No	Yes	Yes	No	Yes	Yes	No	Yes	Yes	No	8 (53)
GA at delivery,^d^ median (range), weeks	37^6/7^	19^0/7^	19^0/7^	22^0/7^	38^0/7^	16^0/7^	35^5/7^	36^4/7^	20^0/7^	38^2/7^	37^1/7^	21^1/7^	31^1/7^	36^1/7^	16^3/7^	31^1/7^ (16^0/7^–38^2/7^)
Fetal hydrops, *n* (%)	No	No	Yes	No	No	Yes	No	Yes	NA	No	No	No	Yes	Yes	Yes	6 (40)
GA at first IUT, median (range), weeks	24^3/7^	NA	18^0/7^	NA	22^0/7^	15^6/7^	19^2/7^	18^5/7^	NA	24^6/7^	19^4/7^	20^5/7^	18^6/7^	26^3/7^	NA	19^4/7^ (15^6/7^–26^3/7^)
IUT(s) per pregnancy, median (range)	5	NA	1	NA	5	1	1	6	NA	5	1	1	5	4	NA	4 (1–6)
RhD alloantibody type, *n* (%)	Yes	Yes	No	Yes	Yes	Yes	Yes	Yes	Yes	Yes	Yes	Yes	Yes	Yes	Yes	14 (93)
IVIG administration, *n* (%)	No	No	No	No	No	Yes	No	No	No	No	No	No	No	No	No	1 (7)

*Note*: Characteristics of pregnant participants with EOS‐HDFN at the time of enrollment in CLARITY, and outcomes of their most recent qualifying pregnancies. Detailed demographic and baseline characteristics are provided in Table S2.

Abbreviations: EOS‐HDFN, early‐onset severe hemolytic disease of the fetus and newborn; GA, gestational age; HDFN, hemolytic disease of the fetus and newborn; IUT, intrauterine transfusion; NA, not available.

^a^ Six participants with anti‐D also presented with ≥1 other alloantibody type, including anti‐C (*n* = 3), anti‐C and ‐E (*n* = 2), and anti‐E (*n* = 1).

^b^ The time between the study pregnancy and the last qualifying pregnancy was defined as the elapsed time in years between delivery in the last qualifying pregnancy and the date of conception of the study pregnancy. The date of conception of the study pregnancy was defined as the date of informed consent minus GA at informed consent.

^c^ The time between the study pregnancy and the last HDFN‐affected pregnancy was defined as the elapsed time in years between delivery in the last HDFN‐affected pregnancy (if the last qualifying pregnancy was not the last HDFN‐affected pregnancy) or the last qualifying pregnancy (if the last qualifying pregnancy was the last HDFN‐affected pregnancy) and the date of conception of the study pregnancy. An HDFN‐affected pregnancy was defined as a pregnancy with an antigen‐positive fetus.

^d^ The time at delivery for either live birth or fetal death.

### 3.2 Antenatal outcomes

Of 15 pregnant participants, 14 (93%; 95% CI, 68.1%–99.8%) pregnancies resulted in a live birth, with GA at delivery ranging from 31^4/7^–38^0/7^ weeks (Table 2 and Figure 2). Of these, seven (50%) were term births (range, 37^0/7^–38^0/7^ weeks), six (43%) were moderate‐late preterm (32^4/7^–35^5/7^), and one (7%) was very preterm (31^4/7^ weeks); 12 (86%) pregnancies resulted in cesarean deliveries, while two (14%) were delivered vaginally. One (7%) fetal death occurred at 16^5/7^ weeks GA followed by a dilation and curettage procedure in a pregnancy with anti‐C and ‐D alloantibodies.

**TABLE 2:** Summary of antenatal outcomes.

Outcome	Most recent qualifying pregnancies (*N* = 15)	Study pregnancies (*N* = 15)
Participants with a live birth at ≥32 weeks GA without an IUT, *n* (%)	0	2 (13)
Participants with a live birth at ≥32 weeks GA, *n* (%)	7 (47)	13 (87)
Participants without an IUT, *n* (%)	4 (27)	2 (13)
Antenatal outcomes		
Participants with a live birth, *n* (%)	8 (53)	14 (93)
GA at delivery, median (IQR), weeks	36^6/7^ (35^6/7^–37^6/7^)	36^2/7^ (33^5/7^–37^2/7^)
Participants with ≥1 IUT, *n* (%)	11 (73)	13 (87)^a^
GA at first IUT, median (IQR), weeks	19^4/7^ (18^5/7^–24^3/7^)	24^0/7^ (18^0/7^–25^3/7^)
IUT(s) per pregnancy, median (IQR)	4 (1–5)	4 (3–5)
GA at delivery,^b^ median (IQR), weeks	31^1/7^ (19^0/7^–37^1/7^)	35^5/7^ (33^0/7^–37^2/7^)
Participants without an IUT before 24 weeks GA, *n* (%)	0	9 (60)
Fetal hydrops, *n* (%)	6 (40)	1 (7)
Participants with ≥1 IVIG administration, *n* (%)	1 (7)	8 (53)
IVIG administrations per pregnancy, median (range)	NA	12.5 (1–20)

Abbreviations: GA, gestational age; IQR, interquartile range; IUT, intrauterine transfusion; IVIG, intravenous immunoglobulin; NA, not available.

^a^ Of the 13 participants with ≥1 IUT, five had ≥1 IUT prior to informed consent, with the time from first IUT to enrollment ranging from 12–77 days; the remaining eight participants had their first IUT after informed consent (Day 1–95).

^b^ The time at delivery for either live birth or fetal death. Median (range) time from informed consent to delivery was 13^0/7^ weeks (0^0/7^–19^0/7^).

**FIGURE 2:**
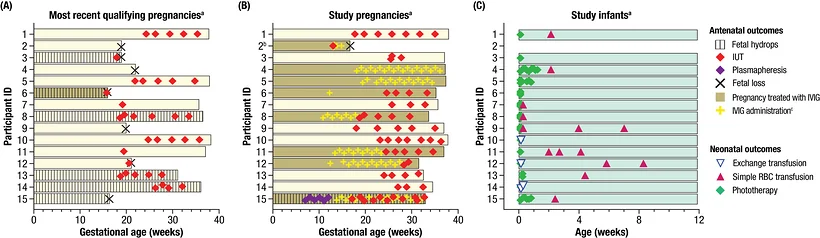
Antenatal outcomes in (A) most recent qualifying pregnancies and (B) study pregnancies by pregnant participant and (C) postnatal outcomes by study infant. Antenatal treatments and outcomes for pregnant participants during their most recent qualifying and study pregnancies, as well as postnatal treatments and outcomes for infants born during the study, are shown. GA, gestational age; IUT, intrauterine transfusion; IVIG, intravenous immunoglobulin; RBC, red blood cell. ^a^Bars for qualifying and study pregnancies extend to GA at delivery and for study infants extend to weeks of the follow‐up. ^b^There was no evidence of fetal hydrops (ascites). ^c^GA at IVIG administration in the most recent qualifying pregnancy was unknown. Details of IVIG administration in study pregnancies are provided in Table S4.

Two participants (13%; 95% CI, 1.7%–40.5%) achieved the primary endpoint of a live birth at ≥32 weeks GA without an IUT; both received IVIG during the study (Participants 4 and 5 from the same study site; starting at 18–19 weeks GA; Tables S3 and S4). Thirteen (87%) pregnant participants received IUTs, with GA at first IUT ranging from 13^0/7^–28^2/7^ weeks. Among the 13 participants, 12 had MCA‐PSV ≥1.5 MoM prior to the first IUT; one underwent IUT despite MCA‐PSV <1.5 MoM with cordocentesis‐confirmed anemia (hemoglobin 76 g/L; hematocrit fraction 0.24; Table S5 and Figure S2), and one had MCA‐PSV ≥1.5 MoM 1 day before IUT but was <1.5 MoM on the day of the procedure (Figure S3). Nine (60%; 95% CI, 32.3%–83.7%) pregnant participants had a live birth without any IUTs before 24 weeks GA. In the pregnancy resulting in fetal death at 16^5/7^ weeks, an IPT was reported at 13^0/7^ weeks GA when MCA‐PSV was 45.92 cm/s, and four IVIGs were administered from 13^1/7^–14^6/7^ weeks GA; the MCA‐PSV was 39.85 cm/s at 13^4/7^ weeks GA. There was no evidence of fetal hydrops (ascites). The participant also had a history of spontaneous miscarriage at 19^0/7^ weeks GA in the most recent qualifying pregnancy.

Compared with the previous most recent qualifying pregnancies, the study pregnancies had fewer fetal hydrops, more live births, later GA at delivery, and more received IVIG (Table 2). Eight (53%) participants received IVIG during the study pregnancies, with a median of 12.5 administrations (range, 1–20) per participant at 0.5–1 g/kg, starting at 10^6/7^–19^3/7^ weeks GA (Table S4).

A post hoc analysis showed that, of the seven pregnant participants who did not receive IVIG, none (0%; 95% CI, 0–41.0) met the outcome of a live birth delivered at ≥32 weeks GA without an IUT. Of the eight pregnant participants who received IVIG, two (25%; 95% CI, 3.2–65.1) met this outcome (Participants 4 and 5); both received ≥15 weekly IVIG administrations. The remaining six pregnant participants who received IVIG had between 1 and 11 total IUTs; all received ≥4 weekly IVIG administrations, except one participant with a single IVIG administration. One (7%) pregnant participant who received IVIG (Participant 15) also underwent six plasmapheresis sessions for anti‑D antibodies as part of local clinical management, with the first occurring at 7 weeks GA (Figure 2B). The participant also underwent 11 serial IUTs, with available pre‐IUT hematocrits ranging from 12%–35% and post‐IUT hematocrit values increasing to 32%–48%; post‐IUT hemoglobin values ranged from 105–120 g/L.

### 3.3 Complications associated with IUT

Among the 13 pregnant participants treated with IUTs (a total of 60 IUTs across participants), four (31%) pregnant participants reported a total of six IUT‐related complications (Table S6). The complications were fetal bradycardia (2 events at 28^5/7^ and 31^5/7^ weeks GA in Participant 1, who delivered at 38^0/7^ weeks GA), cord bleeding and preterm premature rupture of membranes (1 event each at 29^2/7^ weeks GA in Participant 12, who delivered at 31^4/7^ weeks GA), and other (one event related to transfusion in Participant 3 and one event of mild ascites in Participant 14).

### 3.4 Complications associated with IVIG

Among the eight pregnant participants who received IVIG (a total of 92 administrations across participants), three (38%) reported a total of seven IVIG‐related complications (Table S7). The complications were abnormally rapid heart rate (one event in Participant 8), headache and nausea (one event each in Participant 2), and a combination of abnormally rapid heart rate, headache, lower back pain, and “feeling warm” (one event each in Participant 6). One pregnant participant (Participant 12) developed aseptic meningitis associated with the first IVIG administration, resulting in a 3‐week delay in further IVIG treatment.

### 3.5 Birth and postnatal outcomes through 12 weeks

Neonatal baseline characteristics and birth outcomes are summarized in Table S8. All 14 neonates required ≥1 phototherapy session per local guidelines based on hyperbilirubinemia by GA and presence of other neurotoxicity risk factors [28] (Table 3). One or more high‐intensity phototherapy sessions (double bank and/or biliblanket) were administered to 11 (79%) neonates. Three (21%) neonates required ≥1 exchange transfusion (Table 3 and Figure 2B). Nine (64%) required ≥1 simple RBC transfusion during the first 12 weeks of life; of these, three received their first transfusion on Day 2, and six received their first transfusion at ≥14 days of age. One of these six also received an exchange transfusion at birth. Three (21%) required neither exchange nor simple RBC transfusions, one of whom also required no antenatal IUTs, but underwent high‐intensity phototherapy.

**TABLE 3:** Summary of postnatal outcomes.

	Neonates/infants from the most recent qualifying pregnancies (*N* = 8)^a^	Study neonates/infants (*N* = 14)
Phototherapy		
Infants who required ≥1 phototherapy session, *n* (%)	6 (75)	14 (100)
Number of hours of phototherapy, median (range)	NA^b^	116.5 (12.0–216.0)
Exchange transfusion		
Infants who required ≥1 exchange transfusion, *n* (%)	2 (25)	3 (21)
Number of exchange transfusions, median (range)	NA^b^	1 (1–2)
Simple transfusion in the first 3 months of life		
Infants who required ≥1 simple transfusion, *n* (%)	4 (50)	9 (64)
Number of simple transfusions, median (range)	NA^b^	1 (1–3)

*Note*: Postnatal outcomes from neonates/infants born to pregnant participants with EOS‐HDFN.

Abbreviations: EOS‐HDFN, early‐onset severe hemolytic disease of the fetus and newborn; NA, not available.

^a^ Percentages were calculated based on the number of live births.

^b^ Data are not available as the number was not reported in some participants.

Compared with the eight live births from the 15 most recent qualifying pregnancies, more infants in the study received phototherapy and simple transfusions, and a similar proportion received exchange transfusions.

All 14 infants had HDFN‐related hospitalizations, with a median length of stay of 7 days (range, 3–65). One infant (Participant 11) was hospitalized for 65 days (15 days in NICU followed by transfer to a local hospital) due to severe conjugated cholestasis, which affected liver function and was associated with elevated ferritin levels. Thirteen (93%) infants required NICU admission for HDFN‐related treatment, with a median length of stay of 5 days (range, 2–34). The infant requiring 34 days in the NICU was born at 31^4/7^ weeks GA due to preterm premature rupture of membranes following an IUT.

## 4 DISCUSSION

CLARITY extends the evidence base by providing prospective, global, observational data across expert referral centers, focused specifically on EOS‐HDFN with prespecified eligibility criteria and endpoints, and describing contemporary management and outcomes in this rare high‐risk population from 2019–2023, complementing prior retrospective studies from previous time periods over the past 16–20 years. While not designed to establish causal treatment effects, these hypothesis‐generating data demonstrated that most pregnant participants in the CLARITY study (87%) did not achieve the primary outcome of a live birth at ≥32 weeks GA without any IUTs, with most requiring multiple IUTs, often starting at ≤24 weeks GA. Observed complications included HDFN‐related hydrops (7%) and fetal demise (7%); procedure or treatment‐related complications in pregnant participants including IUT‐related bradycardia, cord bleeding, and preterm premature rupture of membranes; and IVIG‐related headache, abnormally fast heart rate, backache, nausea, and aseptic meningitis. Although fetal survival improved in the study pregnancy compared with the participants’ prior qualifying EOS‐HDFN pregnancies, the median number of IUTs per pregnancy remained the same, and nine (60%) pregnancies developed EOS‐HDFN requiring IUT(s) at ≤24 weeks GA.

Outcomes in these study pregnancies with prior histories of EOS‐HDFN are generally consistent with recent retrospective studies that included this high‐risk population where fetal anemia requiring IUTs developed in 88%–100% of pregnancies with the first IUT at a median or mean of 22–23 weeks GA [9, 10, 21, 26]. The rates of fetal hydrops (7%) and fetal loss (7%) reported herein were within the range reported in previous studies, with fetal hydrops reported in 0%–17% and fetal loss in 6%–20% of pregnancies [9, 10, 21]. In these retrospective studies, 13%–22% of neonates had exchange transfusions, which is similar to the 21% observed in neonates in this CLARITY study [9, 10, 21]. NICU stays were required for the majority of neonates, with one experiencing cholestasis and iron overload requiring chelation therapy, a condition associated with hemolysis, especially with multiple IUTs [29, 30].

IVIG is thought to reduce fetal RBC destruction by limiting transplacental transfer and Fc‐mediated activity of pathogenic maternal IgG (via Fc receptor blockade/competition) and also inhibit macrophage Fcγ‐receptor‐driven phagocytosis of antibody‐coated fetal RBCs [31, 32]. In this CLARITY study, among eight participants treated with IVIG, two (25%; 95% CI, 3.2–65.1) met the primary endpoint and delivered after 32 weeks GA without receiving IUTs, whereas the recent retrospective studies reported that 0%–12% of IVIG‐treated, EOS‐HDFN at‐risk pregnant participants achieved this outcome [9, 10, 21]. Although no definitive conclusions can be drawn from these observational findings, these CLARITY results are consistent with prior retrospective data suggesting potential benefit of antenatal IVIG treatment [9, 10, 21]. Evaluation in a randomized controlled trial would be needed to assess the effect of IVIG on the course and severity of the disease in pregnancies at high risk for EOS‐HDFN.

This study demonstrated the substantial challenges associated with current practices for managing EOS‐HDFN in the setting of experienced healthcare centers. The need for multiple IUTs and IVIG administrations can result in serious pregnancy complications and considerable patient burden, highlighting the continuing high unmet medical need for a noninvasive medical intervention for EOS‐HDFN. IVIG dosing regimens varied substantially across centers in this study, consistent with variation in management reported from a large global study [33], and differed from a recently published international expert Delphi consensus, which recommends antenatal IVIG for managing pregnancies with EOS‐HDFN, starting at 10–14 weeks GA with a dose of 1 g/kg per week [23]. IVIG is also associated with the health care system burden (e.g., resource utilization, cost, potential for product shortages) and patient burden (e.g., tolerability issues, long treatment times) [10, 31, 34, 35, 36]. This study, taken together with the previous retrospective studies evaluating off‐label use of IVIG and the UNITY study of nipocalimab [9, 10, 20, 21, 26], support the high unmet medical need with severe fetal and newborn morbidity and mortality with potential for benefit for noninvasive antenatal treatments in high‐risk pregnant individuals with prior histories of EOS‐HDFN. Further evaluation of antenatal IVIG treatment in randomized controlled studies will inform on the potential for improved antenatal and neonatal outcomes.

### 4.1 Strengths and limitations

The strengths of this study include its prospective design aimed at assessing the most current outcomes of high‐risk alloimmunized pregnancies with a history of EOS‐HDFN, which complements recent retrospective studies [9, 10, 21, 26]. The baseline characteristics of the pregnant participants evaluated in CLARITY were generally representative of the rare EOS‐HDFN population, based on rigorous eligibility criteria for the study and previous qualifying pregnancy [15, 25, 37, 38, 39]. The inclusion of anti‐D and anti‐K alloantibody types and the proportions of these major alloantibody types and the associated secondary alloantibodies responsible for the vast majority of EOS‐HDFN pregnancies indicate that this cohort is representative of the majority of high‐risk, high unmet medical need populations across most global regions.

Limitations include small sample size due to the rarity of EOS‐HDFN (resulting in wide Clopper–Pearson 95% CIs), nonrandomized design, and overlap of two participants also included in one published retrospective study and the meta‐analysis [21, 26]; therefore, these outcome estimates should be interpreted with caution. While management and outcomes were likely representative of standard of care at the global expert HDFN referral centers, variability in management (e.g., protocol for confirmation of fetal antigen positivity, MCA Doppler thresholds for intervention, IUT techniques, IVIG dosing regimens, and postnatal treatments) may lead to occasional misclassification, complicate pooled interpretation, and limit generalizability to other referral centers or regions with differences in local guidelines. Potential selection bias may have occurred as 78% of centers also participated in UNITY and contributed 47% of participants. However, CLARITY, UNITY, and prior retrospective studies enrolled comparable high‐risk populations, each requiring prior EOS‐HDFN with onset at ≤24 weeks GA [9, 10, 20, 21, 26]. Baseline alloantibody profiles were also similar across studies (anti‐D 70%–80%; anti‐K 18%–24%). The enrollment period from ≥8 weeks GA to delivery may also result in survival bias. To enrich the population selected in UNITY, the more restrictive criteria were selected (e.g., hemoglobin <0.55 MoM, exclusion of preeclampsia) and also applied to CLARITY, which may limit generalizability and comparability with studies using different criteria. Comparisons between outcomes in most recent qualifying and study pregnancies are limited since pregnancies were not all managed at the same HDFN referral center. Nevertheless, the overall management and HDFN outcomes are comparable to the prior retrospective studies [9, 10, 21, 26], suggesting that this cohort may supplement and reinforce the understanding of outcomes in this rare and high unmet need population.

## 5 CONCLUSION

This prospective, global, observational study of pregnancies affected by EOS‐HDFN reports outcomes generally consistent with previous retrospective studies, indicating a high risk of early IUT, neonatal transfusions, and fetal/neonatal morbidity and mortality [9, 10, 21, 26]. Almost all pregnant participants required multiple IUTs (both IPT and IVT), often starting at ≤24 weeks GA, with approximately 30% of participants experiencing complications related to these procedures. The affected neonates continued to experience hemolytic anemia requiring neonatal transfusions, prolonged hospitalizations, and NICU hospitalizations. The effect of antenatal IVIG treatment on the course and severity of EOS‐HDFN warrants evaluation in a larger prospective randomized controlled trial. The results underscore the high unmet medical need for effective and noninvasive treatments for EOS‐HDFN.

## AUTHOR CONTRIBUTIONS

**Dick Oepkes**: Conceptualization; investigation; methodology; supervision; writing—reviewing and editing. **Kenneth J. Moise Jr**.: Conceptualization; investigation; methodology; supervision; writing—reviewing and editing. **Enrico Lopriore**: Conceptualization; investigation; methodology; supervision; writing—reviewing and editing. **Eleonor Tiblad**: Conceptualization; investigation; methodology; supervision; writing—reviewing and editing. **E. J. T. (Joanne) Verweij**: Conceptualization; investigation; methodology; supervision; writing—reviewing and editing. **Rory Windrim**: Investigation; writing—reviewing and editing. **Pranav Pandya**: Investigation; writing—reviewing and editing. **Robert M. Silver**: Investigation; writing—reviewing and editing. **Stephen P. Emery**: Investigation; writing—reviewing and editing. **John Smoleniec**: Investigation; writing—reviewing and editing. **Olga Ocón‐Hernández**: Investigation; writing—reviewing and editing. **Rachel K. Morris**: Investigation; writing—reviewing and editing. **Russell S. Miller**: Investigation; writing—reviewing and editing. **James B. Streisand**: Conceptualization; methodology; supervision; writing—reviewing and editing. **Diane D. Harrison**: Investigation; writing—reviewing and editing. **Valerie Smith**: Data curation; formal analysis; validation; writing—reviewing and editing. **Lisa B. Schwartz**: Investigation; methodology; supervision; analysis; validation; writing—reviewing and editing. **May Lee Tjoa**: Conceptualization; data curation; formal analysis; investigation; methodology; supervision; validation; writing—reviewing and editing. **Leona E. Ling**: Conceptualization; data curation; formal analysis; investigation; methodology; supervision; validation; writing—reviewing and editing. **Shumyla Saeed‐Khawaja**: Conceptualization; data curation; formal analysis; investigation; methodology; supervision; validation; writing—reviewing and editing. **Yosuke Komatsu**: Conceptualization; data curation; formal analysis; investigation; methodology; supervision; validation; writing—reviewing and editing.

## CONFLICT OF INTEREST STATEMENT

D.O., E.L., and E.T. received consulting fees for membership on steering committees and advisory boards for clinical studies from Momenta Pharmaceuticals, Inc., and Johnson & Johnson. K.J.M. served as the overall principal investigator for the Phase 2 trial of nipocalimab (UNITY); received funding from Momenta Pharmaceuticals, Inc., paid on his behalf to the McGovern Medical School—UT Health and from Johnson & Johnson paid on his behalf to Dell Medical School at The University of Texas at Austin for a clinical trial on a monoclonal antibody for the treatment of hemolytic disease of the fetus and newborn; served on the steering committees and advisory boards for clinical studies for Momenta Pharmaceuticals, Inc., and Johnson & Johnson but has not received funding for these activities; receives royalty funding from UpToDate, Inc., for authorship on various chapters, consulting fees from Health Management Associates, Inc., for consultation on the formation of fetal centers, consulting fees from BillionToOne, Inc., paid on his behalf to Dell Medical School at The University of Texas at Austin, and honoraria from GLC Healthcare, Inc., for podcast content on hemolytic disease of the fetus and newborn; and serves as a nonpaid consultant for Immunology for Johnson & Johnson. E.J.T.V. serves as the principal investigator of the UNITY, CLARITY, and AZALEA studies in The Netherlands. R.K.M. is the principal investigator for the AZALEA study in the United Kingdom and serves on the steering committee for the trial and received consulting fees for membership on steering committees and advisory boards for clinical studies from Momenta Pharmaceuticals, Inc., and Johnson & Johnson. R.S.M. received consulting fees from Johnson & Johnson; serves as a chapter author for UpToDate, Inc., on an unrelated topic; and receives royalty funding from UpToDate, Inc. J.B.S. was a paid consultant to Momenta Pharmaceuticals, Inc., while providing clinical development services (protocol design, study oversight, medical monitoring) for this study. D.D.H. is a consultant whose work was funded by Johnson & Johnson and holds stock/stock options from Johnson & Johnson. V.S., L.B.S., M.L.T., L.E.L., S.S.‐K., and Y.K. are employees of Johnson & Johnson and hold stock/stock options from Johnson & Johnson. R.W., P.P., R.M.S., S.P.E., J.S., and O.O.‐H. declare no conflicts of interest.

## ETHICS STATEMENT

This study was approved by the appropriate institutional review board/independent ethics review committees at each site (details are provided in Table S1).

## Supporting information



Supporting Information

## Data Availability

Although these data are not currently publicly available for sharing, requests for sharing can be sent to the corresponding author and will be evaluated on an individual basis.
