# Supplementary material for: Characterization of the clinical course of early‐onset severe hemolytic disease of the fetus and newborn
Source: Pregnancy (Hoboken). 2026 Jul 12;2(4):e70352. doi: 10.1002/pmf2.70352 (PMC13624847; doi:10.1002/pmf2.70352)
Supplement: Supplementary file 1 [file PMF2-2-e70352-s001.docx]

# Supporting Information

**Characterization of the clinical course of early-onset severe hemolytic disease of the fetus and newborn**

Figure S1. CLARITY study design

Figure S2. Scatter plot of fetal hemoglobin results at the time of first IUT by gestational age

Figure S3. MCA-PSV from baseline to first IUT

Table S1. List of Institutional Review Boards and Ethics Committees

Table S2. Demographics and Baseline Characteristics of Pregnant Participants and Their Qualifying Pregnancies

Table S3. Summary of GA at First IUT and Delivery Based on Outcomes of Interest

Table S4. IVIG Administration Information During Pregnancies

Table S5. Individual Fetal Hemoglobin/Hematocrit Prior to the First IUT

Table S6. Proportion of IUTs With Complications in Pregnant Participants

Table S7. Proportion of IVIG Administrations with Complications in Pregnant Participants

Table S8. Summary of Baseline Characteristics and Birth Outcomes in Neonates

# Figure S1. CLARITY study design

EGA, estimated gestational age; EOS-HDFN, early-onset severe hemolytic disease of the fetus and newborn; GA, gestational age; SOC, standard-of-care.

^a^Participants who were pregnant at a GA of ≥8 weeks were screened for inclusion during the current pregnancy and enrolled in the study at any time during the current pregnancy prior to delivery.

^b^Treatments received for EOS-HDFN per SOC up to 3 months of age and outcomes of interest through 1 year.

# Figure S2. Scatter plot of fetal hemoglobin results at the time of first IUT by gestational age

IUT, intrauterine transfusion; MoM, multiples of median.

Mild fetal anemia: 0.84-0.65 MoM; moderate fetal anemia: <0.65-0.55 MoM; severe fetal anemia: <0.55 MoM (Mari et al. N Engl J Med. 2000;342(1):9-14).

# Figure S3. MCA-PSV from baseline to first IUT^a^

IUT, intrauterine transfusion; MCA-PSV, peak systolic velocity in the middle cerebral artery; MoM, multiples of median.

^a^Slope regression line is based on values for all participants

# Table S1. List of Institutional Review Boards and Ethics Committees

| **Country/Territory** | **IRB/EC Name/Address** | **Site** |
| --- | --- | --- |
| United States | University of Cincinnati Institutional Review Board  University Hall, Suite 300, 51 Goodman Drive  Cincinnati, OH, 45221-0567 United States | Site 1002 - University of Cincinnati Health Primary Care  231 Sabin Way  Cincinnati, OH, 45267 United States |
| United States | Advarra  6940 Columbia Gateway Drive, Suite 110  Columbia, MD, 21046 United States | Site 1003 - UPMC Magee-Womens Hospital 300 Halket Street  Pittsburgh, PA, 15213 United States |
| United States | Columbia University Institutional Review Boards  154 Haven Avenue. 1^st^ Floor  New York, NY, 10032 United States | Site 1004 - Columbia University Medical Center 622 West 168th Street  New York, NY, 10032 United States |
| United States | University of Utah Institutional Review Board  Research Administration Building, 75 South 2000 East  Salt Lake City, Utah, 84112 United States | Site 1005 - University of Utah Health 50 North Medical Drive  Salt Lake City, UT, 84132 United States |
| United States | Oregon Health and Science University (OHSU) IRB  0690 SW Bancroft  Portland, Oregon, 97239-4244 United States | Site 1007 - Oregon Health and Science University  Dept. of Obstetrics and Gynecology 3303 Southwest Bond Avenue Portland, OR, 97239  United States |
| Canada | Mount Sinai Hospital Research Ethics Board 700 University Avenue, Suite 8-600 Toronto, ON, M5G 1Z5  Canada | Site 2001 - Mount Sinai Hospital – Canada 600 University Avenue  Toronto, ON, M5G 1X5 Canada |
| Canada | Research Ethics Board of the CHU Sainte- Justine  3175, Chemin de la Cote-Sainte-Catherine  Montreal, QC, H3T 1C5  Canada | Site 2002 - Centre Hospitalier Universitaire Sainte-Justine  3175 Chemin de la Côte-Sainte-Catherine Montréal, QC, H3T 1C5  Canada |
| Canada | University of British Columbia / Children’s & Women’s Health Centre of British Columbia Research Ethics Board  950 West 28^th^ Avenue, Room A2-141A Vancouver, BC, V5Z 4H4  Canada | Site 2003 - British Columbia Women's Hospital & Health Centre  4500 Oak Street Vancouver, BC, V6H 3N1 Canada |
| Netherlands | Dutch Clinical Research Foundation Kwikstaartlaan 42, Unit A0740 3704 GS Zeist  The Netherlands  ---  Medical Ethics Committee Leiden, The Hague, Delft Secretariaat METC LDD  Postzone P5-P  Postbus 9600  2300 RC Leiden The Netherlands | Site 3001 - Leiden University Medical Center Albinusdreef 2  Leiden, Zuid-Holland, 2333 ZA Netherlands |
| Belgium | Ethics Committee Research UZ/KU Leuven Herestraat 49  B 3000 Leuven Belgium | Site 3101 – University Hospitals Leuven Campus Gasthuisberg  Herestraat 49  Leuven, Flemish Brabant, 3000 Belgium |
| Germany | Justus-iebig University Giessen Ethics Committee of Medicine Faculty 11 Klinikstrasse 29  3585 Gießen Germany | Site 3202 - Justus Liebig University Giessen Institute for Clinical Immunology and Transfusion Medicine  7 Langhansstraße  Giessen, Hessen, 35392 Germany |
| Spain | Clinical Research Ethics Committee of the Barcelona Clinical Hospital  Villarroel 170  08036 Barcelona Spain | Site 3401 – Barcelona Clinical Hospital 1 Sabino Arana Street  Barcelona, Barcelona, 08028 Spain |
| Spain | Coordinating Ethics Committee for Biomedical Research in Andalusia  Avda. de la innovación s/n  Edificio Arena 1  41020 Sevilla Spain | Site 3402 - San Cecilio University Hospital Dr. Oloriz Street  Granada, Granada, 18012  Spain |
| Sweden | Regional Ethical Review board in Stockholm Tomtebodavägen 18 A, floor 3  171 65 SOLNA, Stockholm Sweden | Site 3501 - Karolinska University Hospital Center For Fetal Medicine  K2 Medicin, Solna, K2 KEP Stephansson, 171 77 Stockholm  Sweden |
| United Kingdom | North of Scotland Research Ethics Committee Summerfield House  2 Eday Road Aberdeen AB15 6RE  Scotland | Site 3601 - Birmingham Community Healthcare NHS Foundation Trust  Mindelsohn Way  Birmingham, England, B15 2TG United Kingdom |
| United Kingdom | North of Scotland Research Ethics Committee Summerfield House  2 Eday Road Aberdeen AB15 6RE Scotland | Site 3603 - University College London Hospitals NHS Foundation Trust  235 Euston Road  London, England, NW1 2BU United Kingdom |
| Australia | SouthWestern Sydney Local Health District (SWSLHD) Human Research Ethics Committee  Locked Bag 7103  Liverpool BC NSW 1871, Sidney Australia | Site 7001 - Liverpool Hospital Feto-Maternal Unit  Corner of Elizabeth and Goulburn Streets Liverpool NSW 2170, Sidney  Australia |
| Australia | The Royal Women's Hospital Ethics Committee  Locked Bag 300  Cnr Grattan & Flemington Rd, Parkville VIC 3052  Australia | Site 7002 - The Royal Women's Hospital – Parkville  20 Flemington Road  Parkville, VIC, 3052 Australia |

# Table S2. Demographics and Baseline Characteristics of Pregnant Participants and Their Qualifying Pregnancies

| Characteristic | Pregnant participants (N=15) |
| --- | --- |
| **Demographic characteristics at enrollment** |  |
| Age, median (range), years | 36.0 (29-42) |
| Race, n (%) |  |
| Asian | 1 (7) |
| Black or African American | 1 (7) |
| White | 11 (73) |
| Unknown/not reported | 2 (13) |
| Country of study site, n (%)^a^ |  |
| Australia | 1 (7) |
| Canada | 1 (7) |
| The Netherlands | 4 (27) |
| Spain | 1 (7) |
| Sweden | 2 (14) |
| United Kingdom | 2 (14) |
| United States | 3 (21) |
| Alloantibody type, n (%) |  |
| RhD^b^ | 14 (93) |
| Kell | 1 (7) |
| GA at informed consent, median (range), weeks | 22^3/7^ (13^0/7^-35^5/7^) |
| Time between study pregnancy and last qualifying pregnancy,^c^ median (range)^a^ | 3.0 (0.3-14.6) |
| Time between study pregnancy and last HDFN-affected pregnancy,^d^ median (range)^a^ | 2.6 (0.3-14.6) |
| **Most recent qualifying pregnancy outcomes** |  |
| Participants with a live birth, n (%) | 8 (53) |
| Participants with ≥1 IUT, n (%) | 11 (73) |
| GA at first IUT, median (range), weeks | 19^4/7^ (15^6/7^-26^3/7^) |
| IUTs per participant, median (range) | 4 (1-6) |
| GA at delivery,^e^ median (range), weeks | 31^1/7^ (16^0/7^-38^2/7^) |
| Fetal hydrops, n (%) | 6 (40) |
| GA at first IUT, median (range), weeks | 19^4/7^ (15^6/7^-26^3/7^) |
| IUT(s) per pregnancy, median (range) | 4 (1-6) |
| RhD alloantibody type, n (%) | 14 (93) |
| IVIG administration, n (%) | 1 (7) |
| Neonates with phototherapy, n (%)^f^ | 6 (75) |
| Neonates with exchange transfusion, n (%)^f^ | 2 (25) |
| Neonates with simple transfusion, n (%)^f^ | 4 (50) |

Characteristics of pregnant participants with EOS-HDFN at the time of enrollment in CLARITY, and outcomes of their most recent qualifying pregnancies.

EOS-HDFN, early-onset severe hemolytic disease of the fetus and newborn; GA, gestational age; HDFN, hemolytic disease of the fetus and newborn; IUT, intrauterine transfusion.

^a^Percentages were calculated based on the number of pregnant participants with available data (n=14).

^b^Six participants with anti-D also presented with ≥1 other alloantibody type, including anti-C (n=3), anti-C and -E (n=2), and anti-E (n=1).

^c^The time between the study pregnancy and the last qualifying pregnancy was defined as the elapsed time in years between delivery in the last qualifying pregnancy and the date of conception of the study pregnancy. The date of conception of the study pregnancy was defined as the date of informed consent minus GA at informed consent.

^d^The time between the study pregnancy and the last HDFN-affected pregnancy was defined as the elapsed time in years between delivery in the last HDFN-affected pregnancy (if the last qualifying pregnancy was not the last HDFN-affected pregnancy) or the last qualifying pregnancy (if the last qualifying pregnancy was the last HDFN-affected pregnancy) and the date of conception of the study pregnancy. An HDFN-affected pregnancy was defined as pregnancy

with an antigen-positive fetus.

^e^The time at the delivery for either live birth or fetal death.

^f^Percentages were calculated based on the number of live births (n=8).

# Table S3. Summary of GA at First IUT and Delivery Based on Outcomes of Interest

|  | Total  (N=15) | GA at first IUT, median (range), weeks | GA at delivery, median (range), weeks |
| --- | --- | --- | --- |
| Participants with live birth ≥32 weeks GA without an IUT, n (%) | 2 (13) | – | Week 37^2/7^ (37^2/7^-37^3/7^) |
| Participants with live birth <32 weeks GA without an IUT, n (%) | 0 | – | – |
| Participants with live birth without an IUT, n (%) | 2 (13) | – | Week 37^2/7^ (37^2/7^-37^3/7^) |
| Participants with live birth and ≥1 IUT, n (%) | 12 (80) | Week 24^1/7^ (17^1/7^-28^2/7^) | Week 35^3/7^ (31^4/7^-38^0/7^) |
| Participants with fetal death without IUT, n (%) | 0 | – | – |
| Participants with fetal death and ≥1 IUT, n (%) | 1 (7) | Week 13^0/7^ | Week 16^5/7^ |
| Participants without antenatal and postnatal transfusion, n (%) | 1 (7) | – | Week 37^3/7^ |

GA, gestational age; IUT, intrauterine transfusion.

# Table S4. IVIG Administration Information During Pregnancies

| Participant ID | GA, week | Total dose administered^a^ |
| --- | --- | --- |
| 2 | 13^1/7^ | 20 g |
|  | 13^2/7^ | 20 g |
|  | 13^6/7^ | 20 g |
|  | 14^6/7^ | 65 g |
| 4 | 18^1/7^ | 1 g/kg |
|  | 19^1/7^ | 70 g |
|  | 20^1/7^ | 70 g |
|  | 21^1/7^ | 70 g |
|  | 22^1/7^ | 70 g |
|  | 23^1/7^ | 70 g |
|  | 24^1/7^ | 70 g |
|  | 25^1/7^ | 70 g |
|  | 26^1/7^ | 70 g^b^ |
|  | 27^1/7^ | 70 g |
|  | 28^1/7^ | 70 g |
|  | 29^1/7^ | 70 g |
|  | 30^1/7^ | 70 g |
|  | 31^1/7^ | 70 g |
|  | 32^1/7^ | 70 g |
|  | 33^1/7^ | 70 g |
|  | 34^1/7^ | 10 g |
|  | 34^1/7^ | 60 g |
|  | 35^1/7^ | 70 g |
|  | 36^1/7^ | 70 g |
| 5 | 19^3/7^ | 70 g |
|  | 20^3/7^ | Data not listed |
|  | 22^3/7^ | 70 g |
|  | 23^3/7^ | Data not listed |
|  | 24^3/7^ | 70 g |
|  | 25^3/7^ | 70 g |
|  | 26^3/7^ | Data not listed |
|  | 27^3/7^ | 70 g |
|  | 28^3/7^ | Data not listed |
|  | 29^3/7^ | Data not listed |
|  | 30^3/7^ | 70 g |
|  | 31^3/7^ | 70 g |
|  | 32^3/7^ | 70 g |
|  | 33^3/7^ | 70 g |
|  | 34^3/7^ | Data not listed |
| 6 | 12^2/7^ | 57.5 g |
| 8 | 10^6/7^ | 47.5 g |
|  | 11^6/7^ | 47.5 g |
|  | 12^6/7^ | 47.5 g |
|  | 13^6/7^ | 47.5 g |
|  | 14^6/7^ | 47.5 g |
|  | 15^6/7^ | 47.5 g |
|  | 16^6/7^ | 47.5 g |
|  | 17^6/7^ | 47.5 g |
| 11 | 13^3/7^ | 0.5 g/kg |
|  | 14^3/7^ | 0.5 g/kg |
|  | 15^3/7^ | 0.5 g/kg |
|  | 16^4/7^ | 0.5 g/kg |
|  | 17^3/7^ | 0.5 g/kg |
|  | 18^3/7^ | 0.5 g/kg |
|  | 19^3/7^ | 0.5 g/kg |
|  | 20^3/7^ | 0.5 g/kg |
|  | 21^3/7^ | 0.5 g/kg |
|  | 22^3/7^ | 0.5 g/kg |
|  | 23^3/7^ | 0.5 g/kg |
| 12 | 12^3/7^ | 61 g |
|  | 15^2/7^ | 61 g |
|  | 16^3/7^ | 63 g |
|  | 17^3/7^ | 64 g |
|  | 18^3/7^ | 64 g |
|  | 19^3/7^ | 64 g |
|  | 20^3/7^ | 64 g |
|  | 21^3/7^ | 66 g |
|  | 22^3/7^ | 67 g |
|  | 23^3/7^ | 70 g |
|  | 24^3/7^ | 70 g |
|  | 25^3/7^ | 70 g |
|  | 26^3/7^ | 72.5 g |
|  | 27^3/7^ | 70 g |
| 15 | 13^6/7^ | 55 g |
|  | 14^6/7^ | 55 g |
|  | 15^6/7^ | 55 g |
|  | 16^6/7^ | 55 g |
|  | 17^6/7^ | 55 g |
|  | 18^6/7^ | 55 g |
|  | 19^6/7^ | 55 g |
|  | 20^6/7^ | 55 g |
|  | 21^6/7^ | 55 g |
|  | 22^6/7^ | 55 g |
|  | 23^6/7^ | 55 g |
|  | 24^6/7^ | 55 g |
|  | 25^6/7^ | 55 g |
|  | 26^6/7^ | 55 g |
|  | 27^6/7^ | 55 g |
|  | 28^6/7^ | 55 g |
|  | 29^6/7^ | 55 g |
|  | 30^6/7^ | 55 g |
|  | 31^6/7^ | 55 g |

GA, gestational age.

^a^IVIG doses are presented as reported by study sites (g/kg per infusion or g per infusion); participant weights were not collected in this study; therefore, weight-based dosing (g/kg) could not be calculated when doses were reported in grams.

^a^Reported as “mg” due to a typographical error in the database.

# Table S5. Individual Fetal Hemoglobin/Hematocrit Prior to the First IUT

| **Participant ID** | **GA at first IUT** | **Fetal hemoglobin prior to IUT (g/L)** | **Fetal hematocrit fraction prior to IUT** |
| --- | --- | --- | --- |
| 1 | 17^5/7^ | 32.0 | 0.10 |
| 2 | 13^0/7^ | NA | NA |
| 3 | 25^3/7^ | NA | 0.25 |
| 4^a^ | - | - | - |
| 5^a^ | - | - | - |
| 6 | 24^4/7^ | 59.6 | 0.18 |
| 7 | 25^5/7^ | 75.7 | 0.24 |
| 8 | 18^5/7^ | 33.8 | 0.11 |
| 9 | 18^0/7^ | 41.9 | 0.13 |
| 10 | 23^1/7^ | 68.0 | 0.21 |
| 11 | 24^3/7^ | 53.0 | 0.17 |
| 12 | 28^2/7^ | 61.0 | 0.20 |
| 13 | 24^0/7^ | 25.0 | 0.08 |
| 14 | 27^0/7^ | 40.0 | 0.12 |
| 15 | 19^5/7^ | 56.0 | 0.18 |

GA, gestational age; IUT, intrauterine transfusion; NA, not available.

^a^Participant did not receive any IUT during study pregnancy.

# Table S6. Proportion of IUTs With Complications in Pregnant Participants

|  | **Total**  **(N=15)** |
| --- | --- |
| Participants with ≥1 IUT, n (%) | 13 (87) |
| Total number of IUTs administered | 60 |
| Participants with ≥1 IUT complications, n (%)^a,b^ | 4 (31) |
| Fetal bradycardia^c^ | 2 (15) |
| Cord bleeding^d^ | 1 (8) |
| Preterm premature rupture of membranes^d^ | 1 (8) |
| Other^e^ | 2 (15) |

IUT, intrauterine transfusion.

^a^Percentages were calculated based on the number of participants who received ≥1 IUT (n=13).

^b^Some participants reported >1 IUT complication.

^c^Two episodes of fetal bradycardia were reported in Participant 1 at 28^5/7^ and 31^5/7^ weeks GA. The participant subsequently underwent an IUT at 35^0/7^ weeks GA without complications, and delivered at 38^0/7^ weeks GA.

^d^An event of cord bleeding and preterm premature rupture of membranes occurred at 29^2/7^ weeks GA in Participant 12, who delivered at 31^4/7^ weeks GA without fetal demise.

^e^Reported as “unable to get posttransfusion samples due to the needle migrating after transfusion” in Participant 3 and “mild ascites compatible with a small intraperitoneal transfusion” in Participant 14.

# Table S7. Summary of IVIG Administrations With Complications in Pregnant Participants

|  | **Total**  **(N=15)** |
| --- | --- |
| Participants with ≥1 IVIG administration, n (%) | 8 (53) |
| Total number of IVIG administered | 92 |
| Participants with ≥1 IVIG complications, n (%)^a,b^ | 3 (38) |
| Headache | 2 (25) |
| Tachycardia | 2 (25) |
| Lower back pain | 1 (13) |
| Nausea | 1 (13) |
| Other^c^ | 1 (13) |

IVIG, intravenous immunoglobulin.

Note: Among the 8 pregnant participants who received IVIG, 1 pregnant participant developed aseptic meningitis associated with the first IVIG administration, resulting in a 3-week delay in further IVIG treatment.

^a^Percentages were calculated based on the number of pregnant participants who received ≥1 IVIG (n=8).

^b^Two pregnant participants reported >1 IVIG complication: 1 (Participant 6) with 1 event each of tachycardia, headache, lower back pain, and other during the same IVIG administration; and 1 (Participant 2) with 1 event each of headache and nausea.

^c^Reported as “feeling warm.”

# Table S8. Summary of Baseline Characteristics and Birth Outcomes in Neonates

| **Weight at birth, N** | **14** |
| --- | --- |
| Median (range), kg | 2.8  (1.7-3.7) |
| **Length at birth, N** | **9** |
| Median (range), cm | 47.0  (40-53) |
| **Head circumference at birth, N** | **11** |
| Median (range), cm | 34.0  (30.2-42.5) |
| **GA at birth, N** | **14** |
| Median (range), weeks | 36^2/7^  (31^4/7^-38^0/7^) |
| **1-minute Apgar score, N** | **14** |
| Median (range) | 9  (5-9) |
| Neonates with score <5, n (%) | 0 |
| Neonates with score 5-7, n (%) | 3 (21) |
| Neonates with score 8-10, n (%) | 11 (79) |
| **5-minute Apgar score, N** | **13** |
| Median (range) | 9  (5-10) |
| Neonates with score <5, n (%) | 0 |
| Neonates with score 5-7, n (%) | 2 (15) |
| Neonates with score 8-10, n (%) | 11 (85) |
| **10-minute Apgar score, N** | **9** |
| Median (range) | 10  (8-10) |
| Neonates with score <5, n (%) | 0 |
| Neonates with score 5-7, n (%) | 0 |
| Neonates with score 8-10, n (%) | 9 (100) |

Apgar, appearance, pulse, grimace, activity, and respiration; GA, gestational age.
